# Operationalising Genomic Surveillance for Antimicrobial Resistance in Low- and Middle-Income Countries: A One Health Perspective from Bangladesh

**DOI:** 10.3390/microorganisms14030527

**Published:** 2026-02-25

**Authors:** Khushal Singh, Suparna Mitra

**Affiliations:** Leeds Institute of Medical Research, St James Campus, University of Leeds, Leeds LS9 7TF, UK

**Keywords:** antimicrobial resistance (AMR), low- and middle-income countries (LMICs), genomic surveillance, one health, metagenomics, health inequities

## Abstract

Antimicrobial resistance (AMR) represents a critical global health challenge, with low- and middle-income countries (LMICs) disproportionately affected due to limited surveillance capacity. Advances in microbial genomics offer powerful tools for AMR detection and monitoring; however, translating these technologies into sustainable, policy-relevant surveillance systems in resource-constrained settings remains challenging. This review synthesises current approaches to genomic surveillance of AMR in LMICs and presents Bangladesh as a case study to illustrate how genomic, environmental, and clinical data can be integrated within a One Health framework. We examine key barriers to implementation, including laboratory infrastructure, bioinformatics capacity, data governance, and cross-sector coordination, alongside emerging opportunities for capacity building and regional collaboration. Using Bangladesh as a case study, we highlight practical pathways for embedding genomic surveillance into national AMR strategies, integrating human, animal, and environmental reservoirs of antibiotic resistance. We argue that genomic surveillance can move beyond data generation to inform infection prevention, antibiotic stewardship, and public health decision making when supported by context-appropriate infrastructure and interdisciplinary engagement. By focusing on operational and translational considerations rather than technology alone, this review provides actionable insights for microbiologists, public health practitioners, and policymakers seeking to strengthen AMR surveillance systems in LMICs through a One Health approach.

## 1. Introduction

Antimicrobial resistance has escalated from a looming threat to a present-day global crisis, undermining the foundations of modern medicine. The efficacy of antibiotics, which underpins routine surgeries, cancer chemotherapy, organ transplantation, and the management of infectious diseases, is eroding at an alarming rate [1,2]. While this challenge is universal, its burden is not distributed equally. Low- and middle-income countries (LMICs) experience the most severe health and economic consequences of AMR, a reality shaped by a convergence of systemic vulnerabilities, with antibiotic resistance rates three to four times higher than in high-income countries (HICs) [3]. This section delineates the global scale of the AMR crisis and establishes why it is fundamentally a crisis of development and equity, with LMICs at its core.

### 1.1. Literature Search Strategy and Selection Criteria

To ensure a structured and transparent evidence base, we conducted a targeted literature search of peer-reviewed publications and the key grey literature relevant to genomic AMR surveillance and One Health implementation in LMICs. Searches were performed across major bibliographic databases (e.g., PubMed/MEDLINE, Scopus, Web of Science, and Google Scholar) and complemented by targeted retrieval of policy and surveillance documents from international agencies and monitoring platforms (e.g., WHO/GLASS, WOAH, FAO, and World Bank reports), as well as national AMR strategy and surveillance materials where available.

The search terms combined controlled vocabulary and free-text keywords: (i) AMR (“antimicrobial resistance”, “antibiotic resistance”), (ii) genomics (“genomic surveillance”, “whole-genome sequencing”, “WGS”, “metagenomics”, “shotgun metagenomics”), (iii) implementation context (“LMIC”, “low-resource”, “capacity building”, “bioinformatics”, “laboratory strengthening”, “data systems”), and (iv) One Health and sectoral interfaces (“livestock”, “poultry”, “aquaculture”, “wastewater”, “environment”, “food chain”), with additional geographic filters (e.g., “Bangladesh”, “South Asia”) where relevant.

We prioritised studies that met the following criteria: (1) reported genomic or metagenomic approaches applied to AMR surveillance (clinical, animal, food, or environmental); (2) described implementation models, operational barriers, governance, or capacity-building strategies; and/or (3) provided Bangladesh-relevant empirical evidence or comparable LMIC case studies. We included systematic reviews, scoping reviews, surveillance reports, and high-quality primary studies. We excluded duplicates, articles without sufficient methodological detail, and publications that did not meaningfully address genomic surveillance, AMR surveillance systems, or One Health-relevant transmission pathways. Evidence was synthesised narratively and organised thematically to connect technological choices with implementation constraints and policy implications.

### 1.2. The Global Landscape of AMR: Mortality and Economic Projections to 2050

The human and economic toll of AMR is already immense and is projected to reach catastrophic levels without urgent, coordinated global action. Landmark analyses have provided stark figures that quantify the current and future impact of this crisis. In 2019, bacterial AMR was directly responsible for an estimated 1.27 million global deaths and was associated with a staggering 4.95 million deaths [4]. The global prevalence of AMR surged by 65% between 2000 and 2023 and is anticipated to incur costs of USD 300 billion by 2030 [5]. The highest mortality estimates are concentrated in Asia and Africa, largely due to large populations and insufficient regulatory frameworks [6]. For specific pathogens like *Escherichia coli* and *Klebsiella pneumoniae* (*K. pneumoniae*), antibiotic resistance incidence is projected to reach 77% by 2030 [7].

A critical analysis of these mortality trends reveals a significant demographic shift. While concerted public health efforts have successfully reduced AMR-related deaths in children under five by over 50% between 1990 and 2021, a countervailing trend has emerged in older populations. Over the same period, AMR deaths among adults aged 70 and older surged by more than 80%, a trend projected to accelerate dramatically to a 146% increase by 2050 [8]. This demographic realignment signals a fundamental change in the nature of the AMR threat, evolving from a challenge dominated by common community-acquired infections to one deeply enmeshed within advanced healthcare systems [1,4]. For LMICs, which are simultaneously experiencing rapid demographic aging, this portends a future where already strained healthcare systems will be overwhelmed by complex, costly, and often untreatable infections in their most vulnerable populations.

The economic consequences of AMR are equally devastating. A 2019 analysis estimated the global hospital costs associated with AMR at a median value of USD 693 billion, with an additional USD 194 billion in labour productivity losses [9]. Projections from macroeconomic modelling paint an even more alarming picture. The World Bank estimates that AMR could trigger an additional US 1 trillion in healthcare costs by 2050 and result in annual gross domestic product (GDP) losses ranging from USD 1 trillion to USD 3.4 trillion by 2030 [1].

### 1.3. Why LMICs Are the Epicentre: A Synthesis of Systemic Vulnerabilities

The global statistics on AMR mask profound regional disparities. The highest mortality rates attributable to antibiotic resistance are concentrated in LMICs, particularly in Sub-Saharan Africa and South Asia, which registered death rates of 23.5 and 21.5 per 100,000, respectively, in 2019 [10]. This geographic concentration is the result of a confluence of systemic vulnerabilities that create a fertile environment for the emergence, amplification, and transmission of resistant pathogens. AMR in these regions is a symptom of deeper, interconnected challenges in development, governance, and health systems [10,11,12]

The AMR surveillance in LMICs is primarily coordinated through global frameworks led by the World Organisation for Animal Health (WOAH) and the Food and Agriculture Organization (FAO). These programs aim to track antimicrobial use (AMU) and antibiotic resistance trends to mitigate global health risks, as livestock production is estimated to consume more than two-thirds of medically important Antim worldwide [13].

Data from the WOAH and global health research initiatives reveal a stark divide between LMICs and HICs regarding AMR burden, diagnostic capacity, and regulatory oversight. While global animal antimicrobial use (AMU) declined by 5% between 2020 and 2022, the implementation of containment strategies remains uneven across economic levels [14].

Key systemic vulnerabilities include:

High Burden of Infectious Diseases: LMICs bear most of the global burden of bacterial infections, creating a greater baseline need for antimicrobials and, consequently, higher selective pressure for antibiotic resistance [10,11].

In LMICs, the proportion of bloodstream infections caused by ESBL-producing E. coli is substantially higher; GLASS/SDG indicator data show ranges of 20.3–93.1% in LMICs compared with 5.5–57.2% in HICs [15].

Weak Health Systems: Healthcare infrastructure in many LMICs is poorly functioning and under-resourced. This manifests as a lack of access to quality diagnostics, leading to empirical prescribing, i.e., insufficient infection prevention and control (IPC) measures, and a shortage of skilled healthcare personnel [11]. For instance, formal antimicrobial stewardship (AMS) and IPC programs are present in only 42% and 58% of LMIC settings, respectively, compared to 76% and 89% in HICs [16].

Poverty and Inequality: Poverty is a powerful driver of AMR. It limits access to professional healthcare, forces reliance on informal providers, and compels individuals to purchase cheaper, potentially substandard or falsified, antibiotics without a prescription [17,18]. Overcrowded living conditions facilitate the rapid transmission of infectious diseases [12].

Inadequate Water, Sanitation, and Hygiene (WASH): Lack of access to clean water and sanitation is a critical environmental driver of AMR. Contaminated water sources serve as reservoirs for resistant bacteria and ARGs, facilitating their spread [12,19]

Poor WASH conditions account for an estimated 62% of global deaths from diarrhoea among children under five, creating an “infection-malnutrition cycle” that fuels the demand for antibiotics. For example, in rural Bangladesh, randomized controlled trials proved that physical WASH and nutrition interventions could reduce the prevalence of paediatric antibiotic use by 10–14% and the prevalence of multiple antibiotic courses by 26–35% [20].

Unregulated Antimicrobial Use: In many LMICs, weak regulatory frameworks result in the widespread availability of antimicrobials over the counter. This is compounded by the extensive use of antimicrobials as growth promoters in agriculture and aquaculture [2,11,21].

### 1.4. The Vicious Cycle: How AMR Exacerbates Poverty and Inequality

The relationship between AMR and poverty is bidirectional, creating a vicious cycle that threatens to derail progress toward the Sustainable Development Goals. Just as poverty and systemic weaknesses drive the emergence of antibiotic resistance, drug-resistant infections in turn impose devastating economic burdens that deepen poverty and exacerbate inequality [12,18].

This cycle operates through several mechanisms. First, resistant infections are more difficult and costly to treat, often requiring longer hospital stays and more expensive second- or third-line drugs [4,22]. In health systems dominated by high out-of-pocket (OOP) expenditures, the financial shock of treating a resistant infection can be catastrophic for a household [12,18]. Second, prolonged illness leads to significant productivity losses for both the patient and caregivers [22].

This dynamic creates a fundamental paradox of “access versus excess” that is a core feature of the AMR crisis in LMICs. On one hand, millions die from treatable bacterial infections due to a lack of access to essential antibiotics [23]. On the other hand, the unregulated availability of these same drugs fuels rampant overuse and misuse, accelerating antibiotic resistance [1]. Navigating this delicate balance is one of the most profound challenges for policymakers in LMICs.

Addressing AMR is inseparable from achieving the UN Sustainable Development Goals which includes progress on SDG 3 (Good Health and Wellbeing), SDG 6 (Clean Water and Sanitation), and SDG 1 (No Poverty) is essential to break the poverty–AMR feedback loop and reduce health inequities. The comparative regional burden of AMR is summarized in Table 1.

### 1.5. Antimicrobial Resistance in Global Livestock Production

It is estimated that more than two-thirds of medically important antimicrobials are used in animals raised for food, making them massive reservoirs for the selection and dissemination of antibiotic resistance [13]. Data from the WOAH ANIMUSE database, covering 71% of global animal biomass, indicate that while global animal antimicrobial use (AMU) declined by 5% between 2020 and 2022 (from 102 mg/kg to 97 mg/kg), regional progress remains uneven. Africa reported a 20% reduction, Europe 23%, and the Americas 4%, whereas the Middle East showed a 43% increase associated with rapid production intensification. However, the Middle East contributes only 0.3% of total global animal biomass [14].

Cattle warrant particular attention due to their dominant share of global livestock biomass. Even with lower use intensity compared to pigs or poultry, their scale results in substantial overall antimicrobial volumes. Global modelling estimates that cattle account for approximately 53.5% of total antimicrobial use by mass across major livestock species [24]. Species-level intensity estimates for 2020 report antimicrobial use of 59.6 mg per PCU in cattle compared with 173.1 mg per PCU in pigs, illustrating how large biomass can translate into high aggregate consumption even at moderate mg/kg levels [25].

Systemic vulnerabilities persist in many low- and middle-income countries (LMICs). Approximately 22% of reporting countries continue to use antimicrobials for growth promotion, and 7% report use of highest priority critically important antimicrobials (e.g., colistin, enrofloxacin, fosfomycin) for non-therapeutic purposes [14].

### 1.6. Intensive Poultry and Swine Production

Intensive poultry and swine production can act as major hotspots for multidrug resistant pathogens in resource limited settings, driven by high animal density, antimicrobial use, and gaps in biosecurity. Cattle and dairy systems are also important drivers of antimicrobial exposure in LMIC food animal production. In Bangladesh, a recent survey of smallholder livestock farmers found that only 44% obtained antimicrobials through registered medical practitioners and only nineteen percent followed recommended antimicrobial use guidelines, indicating substantial informal access and suboptimal use [26]. Although national regulations restrict animal antimicrobial prescribing to registered veterinarians, over the counter sales and advice from informal providers are still reported, which can increase unnecessary or inappropriate treatment. Evidence from milk safety studies also suggests downstream food chain exposure, with antibiotic residues detected in seven percent of raw milk samples tested in Chattogram, Bangladesh [27].

In Vietnam, pilot surveillance at slaughter points found that almost all *E. coli* and non-typhoidal *Salmonella* isolated from pigs and chickens were resistant to at least one antimicrobial. Multidrug antibiotic resistance was widespread, with ninety four percent of *E. coli* and eighty nine percent of non-typhoidal *Salmonella* classified as multidrug resistant overall. Importantly, swine were strongly represented in this burden, with multidrug resistant non typhoidal *Salmonella* detected in eighty six percent of pig isolates, while chicken isolates reached ninety five percent, highlighting substantial selection pressure across both production systems [28].

In Uganda, the prevalence of MDR *E. coli* in poultry has been reported as high as 62.7%. An Indonesian study found that chicken slaughterhouse wastewater is a key hotspot for antibiotic resistance. ESBL producing *E. coli* made up 13.8 percent of E coli in the effluent, about twice the level seen in nearby rivers at 6.2 percent [29].

### 1.7. Aquaculture

Asia, which accounts for 90% of global production. In this sector, amphenicols (27.4%), tetracyclines (26.2%), and fluoroquinolones (15.8%) are the leading antimicrobial classes used [14]. The high use of fluoroquinolones is particularly concerning given their “highest priority critically important” status for human medicine [30]. Indiscriminate antibiotic use in tilapia and shrimp ponds often results in microbial dysbiosis, which can paradoxically increase fish susceptibility to severe bacterial infections like *Aeromonas hydrophila*. The economic consequences are staggering; in Bangladesh, “hidden” losses from tilapia mortality—often driven by poor biosecurity and disease—are estimated at USD 875.7 million annually. The risk is compounded by the poor quality of veterinary drugs; a review in Asia and Africa found that 52% of veterinary medicine samples were substandard or falsified, providing sub-therapeutic doses that act as a “training ground” for bacterial resistance [23].

## 2. A National Case Study: Unpacking the Drivers of AMR in Bangladesh

**Figure 1:**
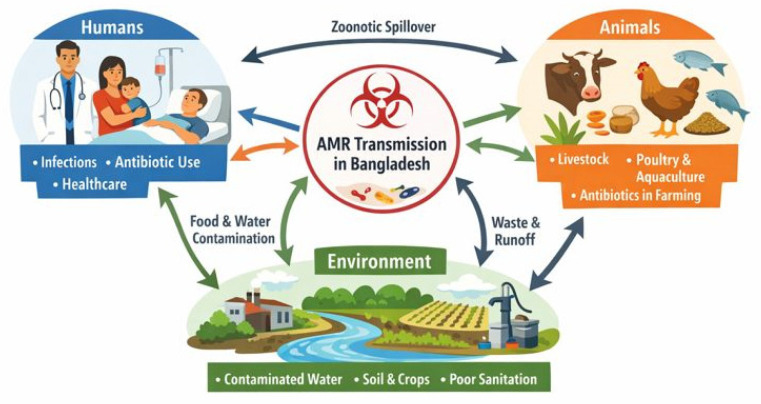
Drivers and ecological vectors facilitating the spread of AMR in Bangladesh. The figure depicts the bidirectional flow of resistant bacteria and genetic elements. Key nodes of transmission include healthcare-associated infections, agricultural overuse of antimicrobials, and environmental pollution, demonstrating how local practices contribute to the maintenance of resistant reservoirs in the ecosystem.

To understand the complex interplay of factors driving AMR in LMICs, a detailed national case study is invaluable. Bangladesh serves as an emblematic case study, offering insights that may be generalisable to other similar settings [1]. Furthermore, as a relatively small country with a more homogenous community structure compared to larger, more diverse nations, findings from targeted studies in Bangladesh can offer insights that are more broadly applicable across its national context, making it a particularly valuable model for understanding AMR dynamics. Its unique combination of rapid urbanisation, a pluralistic and fragmented healthcare system, and an economy heavily reliant on agriculture and aquaculture creates a powerful convergence of drivers for the emergence and spread of resistant pathogens. Applying a One Health perspective, which recognizes the interconnectedness of human, animal, and environmental health. It is essential to fully comprehend the scale of the AMR crisis in Bangladesh (Figure 1).

### 2.1. Socioeconomic and Healthcare Infrastructure: A Fractured System

The foundation of Bangladesh’s AMR problem lies in its socioeconomic context and the structure of its healthcare system. The country is undergoing a rapid and often unplanned urbanisation process, leading to the expansion of densely populated urban slums characterized by inadequate housing, poor sanitation, and contaminated water supplies which induces conditions suitable for the transmission of infectious diseases [31].

A profound disparity exists between urban and rural areas, where over ~60% of the population resides in rural areas [32]. Rural communities face significant barriers to accessing formal healthcare, including a scarcity of qualified doctors, poorly equipped public facilities, and inadequate transportation infrastructure [1,32,33]. This structural inequity is a direct accelerator of AMR. Lacking access to professional medical advice, rural populations are more likely to seek care from informal providers, such as village doctors and local pharmacy owners, who often lack formal training and are known to prescribe antimicrobials irrationally [34,35]. This is exacerbated by a high density of pharmacies (7.2 per 10,000 population), which significantly exceeds that of neighbouring India (5.5 per 10,000) [36].

The healthcare system is overwhelmingly financed by out-of-pocket (OOP) payments, which accounted for 74% of total health expenditure in 2020 [37]. These high out-of-pocket health costs frequently result in catastrophic health expenditure (CHE), a financial burden that disproportionately affects rural households compared to urban residents in Bangladesh [38]. These economic pressures incentivize households—especially in rural settings to bypass formal consultations and obtain antibiotics through lower-cost, more accessible pathways such as drug shops and informal providers, where non-prescription access and incomplete courses are common [39]. This occurs within a broader unregulated, pluralistic system in which antimicrobials are widely available without prescription, undermining stewardship efforts [40].

### 2.2. The One Health Nexus: Interconnected Drivers of Resistance

The drivers of AMR in Bangladesh are not confined to the human health sector but are deeply interwoven with practices in agriculture, aquaculture, and the broader environment, forming a complex One Health nexus.

Human Factors: The practice of self-medication with antibiotics (SMA) is rampant. Studies have documented SMA prevalence rates ranging from 60% to over 88% in various populations [41,42,43]. The primary motivations are the perception that an illness is minor, the desire to save on consultation fees, and reliance on past experiences [44]. This behaviour is enabled by a weak regulatory environment where antibiotics, including potent agents like azithromycin and ciprofloxacin, are widely available over the counter [39,40,44].

Animal production and aquaculture: The animal health sector is a major contributor to the national AMR burden. The country’s vital poultry industry extensively uses antimicrobials for prophylaxis and growth promotion, often without veterinary supervision [34,40]. This has resulted in high levels of multidrug-resistant (MDR) bacteria, such as *E. coli* and *Salmonella*, in poultry products, creating a direct pathway for transmission to humans [45,46]. Similarly, Bangladesh’s burgeoning aquaculture sector is a known hotspot for antibiotic use, contributing to the contamination of aquatic ecosystems [46].

The significant informal trade of live cattle from India, which is nether major hotspot of AMR, further exacerbates AMR risks by undermining sanitary inspections [47].

Environmental Contamination: The confluence of high population density, inadequate sanitation, and intensive agriculture creates a powerful environmental feedback loop for AMR. Untreated sewage, industrial effluent, and farm runoff continuously discharge a cocktail of antibiotic residues, resistant bacteria, and mobile genetic elements into surface waters [11,44,48]. This widespread contamination turns rivers and ponds into reservoirs for AMR, facilitating the transfer of antibiotic resistance genes between environmental and pathogenic bacteria. Shotgun metagenomic studies have confirmed this link, finding that the abundance of ARGs in urban surface waters was strongly correlated with markers of human fecal contamination [49]. Recent environmental surveillance also demonstrates widespread distribution of antimicrobial-resistant enteric bacteria across water sources in Dhaka [50]. Key One Health drivers and gaps contributing to AMR in Bangladesh are summarized in Table 2.

### 2.3. The Clinical Reality: High-Level Antibiotic Resistance in Priority Pathogens

The cumulative impact of these interconnected drivers is reflected in the alarming rates of clinical antibiotic resistance observed in Bangladesh. Data from surveillance and research studies paint a grim picture of rapidly diminishing treatment options. A stark comparison highlights the severity, with one study revealing 89.7% of *E. coli* isolates were resistant to ciprofloxacin, compared to just 11.5% in the UK [51].

*Pseudomonas aeruginosa*: A systematic review (2006–2024) revealed extremely high antibiotic resistance rates, with resistance to ampicillin and amoxicillin exceeding 90%. Only colistin and piperacillin/tazobactam showed lower antibiotic resistance, positioning them as critical last-line therapies [33]. One study found that 100% of *P. aeruginosa* isolates from clinical, environmental, and poultry sources were multidrug-resistant [52,53].

*Klebsiella pneumoniae*: Studies from tertiary care hospitals in Dhaka have found that a majority of *K. pneumoniae* isolates—up to 82% in one study—are MDR (Aminul et al., 2021 [50] Even more concerning is the emergence of extensively drug-resistant (XDR) and pan-drug-resistant (PDR) strains, with one study identifying 22.7% of isolates as XDR and 2.3% as PDR [54].

*Escherichia coli*: As one of the most common causes of infections, high antibiotic resistance rates in *E. coli* are particularly troubling. A study of clinical isolates from Dhaka (2015–2019) found that 96.6% were MDR (Multi-Drug Resistant) [55]. In urinary tract infections, 73.9% of *E. coli* isolates were found to be MDR, with over half showing antibiotic resistance to widely used antibiotics like cefuroxime, ceftazidime, and ciprofloxacin [52].

This clinical reality means that empirical antibiotic therapy is increasingly failing, leading to higher risks of treatment failure, prolonged illness, and mortality.

**Table 2:** Key Drivers of AMR in Bangladesh Across the One Health Spectrum.

Domain	Key Driver	Identified Policy/Practice Gaps	Specific Examples & Data	References
Human Health	Self-medication & Over the Counter (OTC) Access	Weak regulatory enforcement on prescription-only sales; low public awareness and health literacy regarding AMR risks	Prevalence of 60–88% in various populations; key reasons are cost, convenience, and past experiences, Azithromycin and Ciprofloxacin are commonly self-medicated	[40,42,43,44]
	Irrational Prescribing	Lack of standardized treatment guidelines; inadequate antimicrobial stewardship programs (ASPs) in healthcare facilities; limited access to diagnostics	Widespread prescription of antibiotics by unqualified informal providers; pressure on qualified physicians to meet patient expectations	[18,32,40]
Animal Health (Poultry & Livestock) Aquaculture	AMU	Inadequate veterinary services and oversight; lack of enforcement of bans on critically important antimicrobials for non-therapeutic use	Extensive use of antibiotics for growth promotion and prophylaxis in poultry and livestock	[40]
	Food Chain Contamination	Lack of a robust national surveillance system for AMR in the food chain; inadequate food safety regulations and enforcement	High prevalence of MDR *E. coli* (88.3%) and *Salmonella* (75%) in poultry and livestock food products	[32,40]
	Intensive AMU	Paucity of data on the types and quantities of antimicrobials used; lack of specific regulations governing AMU in aquaculture	Direct application of antibiotics to fishponds to control disease in intensive farming systems,	[12,52]
	Environmental Dissemination	Poor integration of aquaculture management into national AMR action plans; lack of environmental monitoring around aquaculture zones	Antibiotic residues and ARGs from aquaculture contaminate surrounding water bodies, contributing to the environmental resistome	[40,52]
Environment	Inadequate Sanitation & Wastewater Treatment	Massive deficit in WASH infrastructure investment; weak enforcement of environmental regulations on industrial and hospital effluent	Untreated or partially treated sewage from urban areas is a primary driver of ARG abundance in surface waters	[1,49]
	Agricultural Runoff	Lack of policies and infrastructure for safe manure management; poor biosecurity practices on farms	Direct disposal of farm waste and manure into the environment pollutes soil and water with resistant bacteria and antibiotic residues	[12,40,56]

## 3. The Promise and Peril of Genomic Surveillance in LMICs

As the scale of the AMR crisis becomes clearer, the limitations of conventional surveillance methods are increasingly apparent. Genomic surveillance, powered by whole-genome sequencing (WGS) and metagenomics, offers a transformative leap in capability. However, its implementation in the resource-constrained settings of LMICs, where it is most needed, is fraught with significant operational, financial, and technical challenges.

### 3.1. Beyond Conventional Methods: The Power of Genomics and Metagenomics for AMR Tracking

Genomic surveillance provides a level of resolution and insight that is unattainable with traditional, culture-based methods. WGS of bacterial isolates allows for a comprehensive characterisation of a pathogen’s genetic makeup, including its lineage, specific AMR determinants, and the mobile genetic elements (such as plasmids) that facilitate horizontal gene transfer [57,58]. By integrating this data with epidemiological information, authorities can detect outbreaks with high precision and untangle complex transmission pathways [58].

Shotgun metagenomics extends this capability by offering a culture-independent approach, sequencing all genetic material within a sample (e.g., sewage, soil). This allows for the characterisation of the entire “resistome,” providing a broad, unbiased picture of the AMR burden in a community or ecosystem and identifying environmental hotspots for AMR dissemination [49,59,60].

### 3.2. Operational Realities: Overcoming Barriers to Implementation

Despite its immense promise, the widespread implementation of genomic surveillance in LMICs faces formidable barriers that span infrastructure, cost, and human resources.

Infrastructural and Financial Barriers: The high upfront and recurring cost of sequencing is a major obstacle, with equipment and reagents often being more expensive in LMICs than in HICs [58]. These technologies also depend on robust supporting infrastructure—such as stable electricity and cold-chain storage—that is often unreliable [11,58]. Financial sustainability is a paramount concern, as many initiatives rely on short-term international grants without secured long-term domestic financing [58,61].

Human Resources and the Bioinformatics Bottleneck: A critical barrier is the shortage of trained personnel, particularly bioinformaticians who can manage and interpret the massive datasets generated by sequencing [57,58]. This “bioinformatics bottleneck” can throttle the translation of raw sequence data into actionable public health intelligence [62].

Data Management and Integration: The value of genomic data is maximized when linked to high-quality clinical and epidemiological metadata. However, data management systems in many LMIC laboratories are fragmented, often relying on paper-based records or non-interoperable electronic systems, hindering the ability to contextualize genomic findings [11,58].

### 3.3. Lessons from the Field: Comparative Analysis of National Genomic Surveillance Programs

Despite these challenges, several LMICs have pioneered the integration of genomics into their national AMR surveillance programs. A comparative analysis of these early efforts, particularly in the Philippines and Cambodia, reveals different strategic models and invaluable lessons. Examples of genomic surveillance implementation models in LMICs are compared in Table 3.

#### 3.3.1. The Philippine Model: Integrated, Decentralized Capacity Building

The Philippines represents an exemplar of integrating WGS directly into a well-established national surveillance network, the Antimicrobial Resistance Surveillance Program (ARSP) [57]. Through a sustained binational collaboration, local capacity for in-country sequencing and data analysis was established at the national reference laboratory. This model yielded significant successes, including the use of genomic data to identify and control a previously undetected, plasmid-driven outbreak of carbapenem-resistant *K. pneumoniae* in a hospital’s neonatal intensive care unit, with a rapid turnaround time of just eight days [57]. However, the program’s success has been heavily dependent on external partnerships, highlighting challenges of long-term financial sustainability and a shortage of skilled bioinformaticians [57,58].

#### 3.3.2. The Cambodian Experience: A Centralized, Pragmatic Approach

Cambodia offers a different, more scalable model for introducing genomic surveillance in a setting with more significant resource constraints. A pilot study successfully supplemented the existing national surveillance system by establishing a centralized sequencing hub paired with cloud-based, open-source bioinformatics platforms [62]. This approach circumvents the need for extensive infrastructure at every site and reduces overall costs [62]. This centralized model has proven effective in identifying clonal outbreaks and tracing community transmission of priority pathogens [62]. The primary challenges remain a heavy reliance on external funding and underlying weaknesses in the foundational surveillance network at sentinel sites [61].

#### 3.3.3. Malaysia: WGS to Reduce Diagnostic Blind Spots

In Malaysia, whole-genome sequencing has been used to study *Salmonella enterica serovar* Typhi in greater detail, helping to track circulating lineages and identify genetic markers linked to antimicrobial resistance [63]. More broadly, genomic surveillance can reveal variation that may affect the performance of molecular diagnostic targets, highlighting the risk of diagnostic escape if assays are not regularly reevaluated against current genomes [64]. This highlights a key lesson for AMR surveillance, that whole-genome sequencing is most effective when combined with routine culture and phenotypic susceptibility testing rather than used as a standalone approach [65].

**Table 3:** Comparison of Genomic Surveillance Implementation Models in LMICs.

Feature	Cambodia	The Philippines	References
Model	Centralized pilot study supplementing the national surveillance system.	Decentralized integration into an established national surveillance program (ARSP).	[61]
Primary Funder/Partner	US National Institute of Allergy and Infectious Diseases and the Bill & Melinda Gates Foundation	Binational collaboration (UK-based institutions) and Philippine Department of Health	[57,62]
Key Successes	Feasibility of centralized sequencing demonstrated; identification of clonal outbreaks (*A. baumannii*) and community transmission clusters (*E. coli*, *Salmonella*)	In-country sequencing and analysis capacity built; rapid (8-day) turnaround for outbreak investigation; detection and control of a plasmid-driven *K. pneumoniae* hospital outbreak	[57,62]
Persistent Challenges	Heavy reliance on external funding; weak foundational laboratory capacity and quality assurance at sentinel sites; sampling bias from urban-centric network	Long-term financial sustainability; supply chain and procurement issues; shortage of skilled bioinformaticians; lack of feedback platforms to sentinel sites	[57,58,61]
Data Management	Cloud-based, open-source bioinformatics solutions; integration with paper-based records at sentinel sites is a challenge	Local bioinformatics server; integration with existing WHONET system	[57,61,62]
Scalability/Sustainability Assessment	More scalable and cost-effective model for initial implementation in low-resource settings, but long-term success depends on strengthening the foundational surveillance network.	High potential for impact but requires significant, sustained domestic investment to overcome reliance on external partners and build a larger skilled workforce.	[61]

## 4. Genomic Surveillance in Practice: The Bangladeshi Context and Comparative Insights

Applying the lessons learned from global and regional experiences with genomic surveillance to the specific context of Bangladesh reveals both immense potential and significant foundational challenges. The country’s fragmented surveillance landscape presents a major hurdle, but emerging metagenomic studies are already beginning to provide unprecedented insights into the national resistome.

### 4.1. Current State of AMR Surveillance in Bangladesh: A Fragmented Picture

Bangladesh’s national AMR surveillance efforts are currently characterized by a bifurcated and fragmented system. On one hand, the Institute of Epidemiology, Disease Control and Research (IEDCR) operates an active, case-based surveillance system across a small network of sentinel sites, aligned with the WHO’s GLASS [34]. This system generates high-quality electronic data but has limited geographic coverage and is not nationally representative [33]. A 2019 study revealed that antibiotic resistance data was available for only 6 out of 64 districts, confirming a lack of comprehensive surveillance [51].

On the other hand, initiatives like the Capturing Data on Antimicrobial Resistance Patterns and Trends in Use in Regions of Asia (CAPTURA) project gather data from a much wider network but suffer from severe data quality issues, with most sites relying on handwritten paper registers [33]. This fundamental disconnects between a small, high-quality system and a large, low-quality one creates a deeply fragmented data landscape with no integrated national platform [33]). While the adoption of the District Health Information System Version 2 (DHIS 2) is a positive step, further enhancements are needed to fully leverage it for AMR policy [33].

### 4.2. Emerging Genomic and Metagenomic Insights from Bangladesh

Despite systemic limitations, pioneering research using genomic and metagenomic approaches is beginning to illuminate the scale and drivers of AMR in Bangladesh.

A landmark study utilized shotgun metagenomics to analyze surface waters and sediments, finding that ARG abundance in some urban samples was 1525-fold higher than in rural sites. Crucially, the study established a strong correlation between ARG abundance and markers of human faecal contamination, identifying untreated sewage as a primary driver of the environmental resistome [49]. Another groundbreaking study applied shotgun metagenomics to the human gut microbiome, revealing that the Bangladeshi gut harbors a significantly higher load and diversity of ARGs compared to other populations, with an average of 75–88 distinct ARGs per sample. Many of these genes were found on mobile genetic elements, indicating a high potential for horizontal gene transfer [66]. Key shotgun sequencing studies in Bangladesh are summarized in Table 4.

### 4.3. Comparative Resistome Analysis: Bangladesh in the Context of Other LMICs

Placing the findings from Bangladesh into a broader context reveals that its challenges are part of a wider pattern observed across many LMICs.

Sewage Metagenomics as a Development Indicator: A large-scale global study analyzing sewage from 74 cities found a clear geographic stratification of the global resistome. The abundance and diversity of ARGs were systematically higher in samples from Africa, Asia, and South America compared to those from Europe, North America, and Oceania [70,71,72]. This disparity correlated most strongly not with antibiotic consumption, but with socioeconomic indicators related to sanitation, health, and governance [70]. This reframes the environmental resistome as a powerful indicator of a country’s public health infrastructure, reinforcing the argument that investments in WASH are a critical AMR mitigation strategy.

AMR in Food Production Systems: Both Southeast Asia and Sub-Saharan Africa are recognized as global hotspots for AMR, driven by the rapid intensification of agriculture and widespread, unregulated antimicrobial use [72,73]. However, a key difference lies in data availability, i.e., research on AMU and AMR in animal production is considerably larger for Southeast Asian countries than for most of Sub-Saharan Africa, where data remains exceptionally scarce [72]. Furthermore, aquaculture represents a uniquely significant driver of AMR in Asia, which accounts for approximately 90% of global production, making it a critical regional priority [74]. 

Comparable shotgun metagenomic sequencing studies across LMICs are summarized in Table 5.

### 4.4. Genomic Surveillance and Policy Interventions: Learning from Other LMICs

Several case studies can be called upon to demonstrate how genomics has directly influenced policy and outbreak control.

Environmental Regulation in Indonesia: A pilot study utilized Oxford Nanopore MinION sequencing to identify chicken slaughterhouse effluent as a “critical control point”. The data revealed an ESBL-to-general E. coli ratio in pre-treated effluent (13.8%) that was double the concentration found in downstream rivers (6.2%), providing the necessary “actionable intelligence” for the government to enforce mandatory multi-stage wastewater treatment systems [29].

Genomic tracking of Streptococcus pneumoniae confirmed that the introduction of the PCV10 vaccine successfully reduced vaccine-type strains from 69.8% to 47.2%. This high-resolution data allowed policymakers to confirm the strategy’s benefit while monitoring for “serotype replacement” by non-vaccine strains [79].

Beyond improving detection, genomic surveillance has directly shaped policy and outbreak control decisions in several countries. Genome sequencing surveys uncovered multiple local outbreaks and plasmid-mediated carbapenem resistance in *K. pneumoniae* that were previously undetected by conventional methods, directly informing infection control practices in Philippine hospitals [80].

Molecular surveillance in neonatal intensive care units in Kenya and Nigeria identified high colonisation rates with ESBL and carbapenemase producers, highlighting the value of active genomic/molecular monitoring to guide antibiotic stewardship and infection prevention policies in LMIC hospital settings [81].

### 4.5. Genomic Surveillance and Policy Interventions: Learning from Other HICs

In the United States, integration of whole-genome sequencing into routine surveillance for carbapenemase-producing organisms allowed public health investigators to refine outbreak definitions and confirm transmission pathways [82]. This enabled resources to be directed towards confirmed clusters while avoiding unnecessary interventions in unrelated cases, demonstrating how genomics can make outbreak responses both more targeted and more efficient [82].

## 5. Synthesis and Strategic Recommendations for a Sustainable Future

The global crisis of antimicrobial resistance is a complex challenge that disproportionately burdens LMICs. As illustrated by the case of Bangladesh, the drivers of AMR are deeply embedded in the socioeconomic fabric of society. Moving forward requires a paradigm shift toward integrated, sustainable strategies that address the root causes of AMR.

### 5.1. Key Insights: Common Themes and Divergent Paths in the Fight Against AMR in LMICs

This review has illuminated several overarching themes. First, AMR is fundamentally a crisis of inequality. Second, the drivers of AMR in LMICs are inextricably linked to broader development challenges, including weak health systems, poverty, and inadequate WASH infrastructure. The One Health framework is an operational necessity for addressing these interconnected pathways. Third, while genomic surveillance holds immense promise, its potential can only be realized if it is implemented strategically and sustainably. Finally, the environmental dimension of AMR is a critical and often-underestimated component of the crisis that demands far greater policy attention.

### 5.2. A Roadmap for Strengthening AMR Surveillance: Integrating Genomics into National Action Plans

To effectively combat AMR, robust surveillance is non-negotiable. Regional partnerships and cross-border collaborations, such as WHO GLASS and SEAR AMR networks, are critical for harmonising genomic data, building capacity, and ensuring that surveillance insights translate into coordinated national and international policies. For LMICs seeking to leverage the power of genomics, a strategic and phased approach is essential.

Recommendation 1 (Foundational): Prioritize Investment in Harmonized Digital Health Infrastructure. Before large-scale genomic surveillance can be effective, the foundational layer of data collection must be strengthened. LMICs should prioritize investment in building unified national AMR databases that can integrate data from diverse sources into a single, accessible platform [33,83].

Recommendation 2 (Strategic): Adopt Context-Appropriate and Scalable Surveillance Models. There is no “one-size-fits-all” model. The centralized hub-and-spoke model piloted in Cambodia represents a more cost-effective and scalable starting point for many LMICs [57,61,62].

Recommendation 3 (Sustainable): Shift Funding and Partnership Models. A shift is needed from short-term, project-based funding toward long-term programmatic support that focuses on creating sustainable local ecosystems. This includes investing in training and retaining local scientists and bioinformaticians and securing dedicated, long-term budget allocations for AMR surveillance [57,58,82].

### 5.3. Policy Imperatives: Addressing Root Drivers Through a One Health Framework

Surveillance alone is insufficient; it must inform policy and action that tackle the underlying drivers of antibiotic resistance.

Recommendation 4 (Governance): Strengthen Regulatory Oversight of Antimicrobial Use. National governments must strengthen and enforce regulations governing the sale and use of antimicrobials. This includes enforcing prescription-only access and banning the use of critically important antimicrobials for growth promotion in livestock [23,49]. Innovative measures, such as color-coded packaging to differentiate antibiotic classes, could also be considered [21].

Recommendation 5 (Investment): Integrate WASH Infrastructure into National AMR Action Plans. The overwhelming evidence from environmental metagenomics demonstrates that poor sanitation and contaminated water are major amplifiers of AMR [36,71]. National Action Plans on AMR must explicitly include major investments in WASH infrastructure as a core intervention.

Recommendation 6 (Prevention): Scale Up Infection Prevention and Control (IPC) and Vaccination. Preventing infections is the most effective way to reduce antimicrobial use. Proven interventions like promoting hand hygiene, ensuring access to safe water, and expanding vaccine coverage can prevent over 750,000 AMR-associated deaths annually in LMICs and must be a cornerstone of any AMR strategy [84].

Recommendation 7 (Engagement): Embed community-driven behaviour change strategies in AMR action plans. Sustainable reductions in inappropriate antimicrobial use require raising public awareness, co-creating solutions with communities, and strengthening health literacy, ensuring that stewardship efforts are culturally appropriate and widely adopted [85].

### 5.4. Future Research Directions: Prioritizing Cost-Effective and Scalable Solutions

Targeted research is needed to guide the efficient allocation of limited resources and develop tools appropriate for LMIC contexts.

Recommendation 8 (Diagnostics): Accelerate Development and Implementation of Affordable Point-of-Care Diagnostics. A major driver of irrational antibiotic use is diagnostic uncertainty. There is an urgent need for research and investment in rapid, affordable, and easy-to-use diagnostic tests that can be deployed at the point of care in low-resource settings [85].

Recommendation 9 (Technology): Leverage Technological Advancements. Emerging tools such as portable sequencing platforms (e.g., Oxford Nanopore MinION) and AI-driven analytics can democratise AMR surveillance by enabling real-time, low-cost genomic analysis, even in remote or resource-limited settings. Future research should prioritise the application of long-read metagenomics for rapid diagnostics and targeted sequencing strategies to enhance surveillance efficiency [86,87]. Developing user-friendly bioinformatics tools remains critical to making complex genomic data more accessible to researchers in LMICs [88].

Recommendation 10 (One Health Metagenomics): Expand Integrated Environmental Surveillance. Building on pioneering studies, integrated metagenomic surveillance should be expanded in sentinel LMICs. Systematically sampling key environmental interfaces—such as urban sewage, agricultural runoff, and retail food products. It can provide a powerful, low-cost method for monitoring the national resistome and identifying emerging threats.

## 6. Conclusions

The AMR crisis is a ticking time bomb, especially for LMICs like Bangladesh, where antibiotic resistance rates are three to four times higher than in HICs. The complex interplay of human, animal, and environmental factors makes understanding and combating AMR exceptionally challenging. Traditional surveillance methods are insufficient to capture this complexity due to fragmented data and systemic weaknesses. Shotgun metagenomic sequencing is therefore not just a novel tool but an essential one for Bangladesh. It bypasses the need for culturing, providing a direct, unbiased snapshot of the entire “resistome” in any given environment, from a patient’s gut to hospital wastewater or a fish farm. This is critical for a country where unregulated antibiotic use in agriculture and aquaculture, combined with inadequate sanitation, creates a vast and interconnected environmental reservoir of antibiotic resistance genes. Pioneering studies in Bangladesh have already demonstrated its power, directly linking the high abundance of ARGs in urban waters to human sewage, providing unequivocal evidence for targeted public health interventions like improving WASH infrastructure.

By embracing shotgun sequencing, Bangladesh can reap substantial public health benefits. It can generate a comprehensive, high-resolution map of its national resistome, identifying hotspots and tracking the transmission of critical antibiotic resistance genes—like *bla*KPC in clinical isolates and *mcr-1* in poultry—across different sectors. This granular data can inform evidence-based policies, guide antimicrobial stewardship, and enable the early detection of emerging threats, such as the discovery of the mobile colistin resistance gene *mcr-5.1* in hospital wastewater. As nation with a relatively homogenous community structure, findings from targeted sequencing studies can be more readily generalized across the country, making Bangladesh a powerful model for developing scalable AMR mitigation strategies that could benefit other LMICs.

Despite its transformative potential, there is a significant lack of shotgun sequencing research in Bangladesh and across most LMICs, creating a critical blind spot in the global understanding of AMR. This gap is largely driven by prohibitive costs, inadequate laboratory infrastructure, and a severe shortage of trained bioinformaticians—the same systemic challenges that fuel the AMR crisis itself. While data from HICs grows, the regions bearing the highest AMR burden remain the most under-studied. The AMR crisis is a global challenge that requires a global response. The evidence is clear: shotgun sequencing provides an unparalleled tool for understanding the complex drivers of antibiotic resistance in hotspots like Bangladesh. Investing in this technology and building local capacity is not merely a national priority for Bangladesh; it is a global public health imperative. By closing the research gap and empowering LMICs with these advanced surveillance tools, the global community can move from reactive crisis management to proactive, data-driven strategies to safeguard the efficacy of modern medicine for future generations.

## Figures and Tables

**Table 1:** Comparative Burden of AMR: LMIC Regions vs. High-Income Countries (HICs).

Metric	Global Average	North America	Europe	South Asia	Sub-Saharan Africa	References
Attributable Deaths per 100,000 (2019)	16.4	13	11.7 (West Europe)	21.5	23.5	[10]
Associated Deaths (millions, 2019)	4.95	~0.3	~0.5 (EU/EEA)	1.07	0.97	[4,8,10]
Projected Attributable Deaths (2050)	1.91 million	Lower Burden	Lower Burden	Highest Burden	Highest Burden	[8,10]
Estimated Annual GDP Loss (by 2030)	USD 1–3.4 trillion	Lower	Lower	High	High	[1]
Third-Gen Cephalosporin-Resistant E. coli Rate (%)	42% (median, 76 countries)	~15%	~15–20%	High (Data Sparse)	High (Data Sparse)	[1]
Methicillin-Resistant S. aureus (MRSA) Rate (%)	35% (median, 76 countries)	~30%	~10–20%	High (Data Sparse)	High (Data Sparse)	[1]

Note: Data for some metrics, particularly antibiotic resistance rates in African and Asian regions, are sparse and aggregated from various sources, reflecting surveillance gaps. Global averages for antibiotic resistance rates are medians reported by WHO GLASS from a subset of countries and may not be fully representative.

**Table 4:** Summary of Key Shotgun Sequencing Studies in Bangladesh.

Source of Sample	Detected Resistance Determinants (Genes/MGEs/Plasmid Replicons)	Implications	Citations
Hospital Wastewater	mcr-5.1; Tn3 transposon	Emergence of colistin resistance in hospital environments	[67]
Gut Microbiome	AmpC (class C) β-lactamase determinants; quinolone resistance determinants	High resistome profile in Bangladeshi population	[66]
Surface Water and Sediments	IncQ1 plasmid replicon associated with sulphonamide resistance	Environmental spread of resistance genes	[49]
Poultry	*bla*_CTX-M-55_, *bla*_CTX-M-65_, *mcr-1*	High levels of MDR *E. coli* in poultry	[33,68]
Goat Meat	*bla*_TEM_, *bla*_SHV_, *bla*_CTX-M_	Transmission of ESBL-producing *Salmonella* via food chain	[69]

**Table 5:** Comparative Shotgun Metagenomic Sequencing Studies Across LMICs.

Pressure Type	Region	Generalisability Factor	Genes + Drug Classes + Mechanisms	Key Findings	Sample Class	Reference
Health	South Asia (Bangladesh, India, Pakistan)	Shared weak healthcare regulation and high non-prescription antibiotic use.	*bla*_TEM_, *strA*, *strB*, *sul2*, *dfrA*	High prevalence of resistance in enteric pathogens from children; over 40 unique ARGs detected.	Clinical (fecal)	[75]
	South Africa	Poor infection control in healthcare settings, similar to other LMICs.	β-lactam (bla family), MLS, tetracycline	36 unique ARGs; prevalent in infants, tied to healthcare exposure.	Clinical (nasopharyngeal)	[76]
Agricultural	South Asia (Bangladesh)	Widespread antibiotic use in poultry/aquaculture, common in many LMICs.	ESBL-associated β-lactamase genes (e.g., *bla*_TEM_)	Higher ESBL-producing coliforms in urban samples; human waste and agriculture linked.	Environmental (surface water, sediments)	[49]
	Southeast Asia (Singapore)	Shared agrarian antibiotic reliance and farm-to-wastewater ARG transfer.	*bla*_KPC-2_, *bla*_CTX-M_, efflux pumps	303 ARG subtypes; high levels from agricultural runoff near healthcare facilities.	Environmental (wastewater)	[77]
Environmen-tal	Sub-Saharan Africa (multiple countries)	Urban sanitation deficits and pollution are common challenges in LMICs.	Macrolide resistance, tetracycline resistance, *bla*_KPC_, *sul* genes	1625 unique ARGs; highest ARG loads in Sub-Saharan Africa, linked to sanitation deficits.	Environmental (urban sewage)	[69]
	East Africa (Kenya)	Contamination of water bodies is a shared issue across many LMICs.	β-lactamase-mediated resistance; vancomycin resistance determinants	9 unique ARGs from 37 high-risk families, indicating environmental health risk.	Environmental (lake water, sediments)	[78]

## Data Availability

This article is a review of previously published studies. No new datasets were generated or analysed during the current study. All data referenced are cited in the reference list.

## Abbreviations

AMR	Antimicrobial Resistance
AMS	Formal Antimicrobial Stewardship
AMU	Antimicrobial Use
ARGs	Antimicrobial Resistance Genes
FAO	Food and Agriculture Organization
GDP	Gross Domestic Product
GLASS	Global Antimicrobial Resistance and Use Surveillance System
HICs	High-Income Countries
IPC	Infection Prevention and Control
LMICs	Low- and Middle-Income Countries
MDR	Multidrug-Resistant
SMA	Self-Medication with Antibiotics
WASH	Water, Sanitation, and Hygiene
WOAH	World Organisation for Animal Health
